# Needs of patients with early psychosis: A comparison of patient’s and mental health care provider’s perception

**DOI:** 10.3389/fpsyt.2022.952666

**Published:** 2022-09-20

**Authors:** P. V. AshaRani, Yeow Wee Brian Tan, Ellaisha Samari, Peizhi Wang, Laxman Cetty, Pratika Satghare, Jayne Ho, Shiyun Astelle Koh, Lee Zhong Yi, Charmaine Tang, Swapna Verma, Mythily Subramaniam

**Affiliations:** ^1^Research Division, Institute of Mental Health, Singapore, Singapore; ^2^Early Psychosis Intervention Programme, Department of Psychosis, Institute of Mental Health, Singapore, Singapore; ^3^Saw Swee Hock School of Public Health, National University of Singapore, Singapore, Singapore

**Keywords:** need assessment, treatment need, care provider, unmet needs, Camberwell Assessment of Needs

## Abstract

**Background:**

Needs define the capacity of a patient to benefit from health care services and a systematic assessment of needs allows planning and delivery of effective treatment to suit patients. This study aimed to understand the (a) needs felt by patients and those perceived by the care providers (CPs), (b) agreement between patients and CPs in the identified needs and (c) factors associated with unmet needs.

**Methods:**

Participants (*N* = 215) were recruited through convenience sampling from the Early Psychosis Intervention Programme (EPIP). Data was captured from patients and CPs using the Camberwell Assessment of Needs Short Appraisal Schedule (CANSAS).

**Results:**

Patients and CPs identified an average of 4.06 and 3.84 needs, respectively. The highest number of unmet needs were identified for the social (50% of patients and CPs) and health domains (31.13% of patients’ vs. 28.30% of CPs). Company, intimate relationships, psychotic symptoms, money, sexual expression and psychological distress, information and benefits were the unmet needs identified by patients, whereas company, intimate relationships, physical health, and daytime activities were identified by CPs. The concordance between patients and CPs was low with majority of the items scoring slight to fair agreement (Cohen’s kappa = 0–0.4). Older age, depression, severe anxiety and having Obsessive-Compulsive Disorder (OCD) were positively associated with unmet needs in patients.

**Conclusion:**

While there was an overall consensus on the total needs and met needs between patients and CPs, the level of agreement between the two groups on various items were low. Different perceptions regarding unmet needs were noted between the groups. A holistic approach that takes into account different facets of the needs of patients together with strategic planning to address unmet needs might improve treatment outcomes and satisfaction.

## Introduction

Schizophrenia and other psychotic disorders are among the top 25 causes of disability worldwide with sufferers recording 13.4 million years of life lived with disability (1, 2). Despite the relatively low prevalence (0.28% in 2016; 1), the disease has tremendous health, social and economic impact. Patients have a higher risk of premature mortality compared to the general population with the gap widening over the years (3). Schizophrenia is also associated with other psychiatric comorbidities, poorer social functioning, and unemployment (4).

Needs are means by which an individual can attain physical, mental, and social wellbeing (5). Maslow described needs through a 5-tier hierarchical pyramidal model where he classified needs as deficiency needs (physiological needs (food, home, sleep, and clothing), need for safety, love and belonging (friendship), and esteem), and growth needs (self-actualization). The model proposed that when a person fulfills his basic needs (deficiency needs), the focus turns toward achieving the higher-level growth needs where they find their meaning in life and life satisfaction (6). In healthcare, needs are described as the capacity of an individual to benefit from the services they receive (7). They get maximum benefit when their needs are met during the treatment process. Healthcare organizations have a key role in this process as they can address and resolve unmet needs and facilitate a smooth reintegration of patients back to their premorbid lives. However, to achieve this aim, organizations need to know what the needs of patients are. This information should be gathered through a systematic needs assessment process that will help identify non-recipients of service, and recipients of ineffective or inappropriate care (7). Such an assessment will allow effective utilization of resources and delivery of patient centric care.

Assessing the needs of patients with chronic mental illnesses is complicated as it varies based on many illness-related factors viz. type of disease, duration, severity, as well as individual psychosocial factors. Patients with schizophrenia have a higher burden of psychiatric comorbidities that are associated with unmet needs and adverse treatment outcomes (8). Depression, anxiety and Obsessive-Compulsive Disorder (OCD) are common psychiatric comorbidities in this group (8). The prevalence of depression varies from 30 to 80% in those with schizophrenia (9). Nearly 38% of patients suffer from anxiety disorder (10) and 12.3% from OCD (11). Given that patients with schizophrenia have diverse needs contributed by the disease characteristics and comorbidities, utmost attention should be paid to various growth and deficiency needs domains to ensure that they get maximum benefit from health care services.

Uygur and Danaci (12) studied the needs of patients with schizophrenia and found that among various health and social needs, those related to psychotic symptoms were unmet in the group. Housework, daily activities, psychological distress, and relationships were also identified as needs. Several studies (13–16) have shown that the needs felt by patients and those perceived by care providers (CPs) often differ. Slade et al. (13) noted that agreement for service satisfaction, as well as help given and received were low between CPs and patients. This has significant implications including treatment non-adherence due to patients’ dissatisfaction with services that fail to address their needs. Joska and Flisher (17) confirmed this by showing that higher fulfilled (met) needs and treatment satisfaction were correlated with better treatment adherence.

Healthcare providers often perceive the needs of their patients based on their clinical expertise and prior training received which may not be reflective of the actual needs of the population. This often leads to a top-down approach in the delivery of care where the patient’s perspective and needs are unintentionally ignored, leading to disengagement from services and unfavorable treatment outcomes (18). Clinicians and policy makers are increasingly acknowledging the treatment gap and social disparities in those with serious mental illnesses and reaching a consensus that a patient centric approach focusing on meeting the needs of patients should be prioritized to achieve better treatment adherence and outcomes (19).

Moreover, there were discrepancies in the social and health needs identified in various studies conducted among those with schizophrenia across the globe. Physical health and money were identified as unmet needs in a Swedish sample of 32 patients (15) while information on conditions/treatment and benefits were identified in a study conducted in Israel [*n* = 52; (19)]. Ochoa et al. (16) investigated a Spanish sample of patients with schizophrenia (*n* = 231) and showed that the unmet needs were mostly related to domains involving psychotic symptoms, company, daytime activities, house upkeep, food, and information. McCrone et al. (20) evaluated the unmet needs in patients with schizophrenia across different countries and showed that unmet needs vary across different countries. The authors reported that unmet needs were high in urban settings which is probably related to the nature of the care delivered and cultural differences. Besides, sociodemographic characteristics influence the perception of unmet needs for mental health care. A study conducted in 2,564 individuals with mental illness showed that unmet needs and contributing factors varied among different sociodemographic groups (21). Women, those with university education and individuals with “white” ethnicity had higher odds of cost related unmet needs while “blacks” tend to report lack of time, information, and transportation (21). The study also identified age as a major determinant of unmet needs. Yang et al. (22) confirmed this observation by showing that age, gender, ethnicity, and education has a role in reporting unmet needs. This calls for more studies in different cultural and geographical contexts to understand the variations in needs among patients with schizophrenia to effectively deliver patient centric care and improve treatment outcomes.

In Singapore, the prevalence of schizophrenia spectrum and other psychotic disorders was 2.3% in the adult population, with an early age of onset (23.1 years) and low treatment gap [80.4% sought treatment, (23)]. The Early Psychosis Intervention Programme (EPIP), a multidisciplinary nationwide program aims to diagnose and manage psychosis at an early stage to improve treatment outcomes and long-term prognosis (24). A program evaluation of EPIP conducted 2 years post implementation showed that 71.1% of patients achieved remission, and 76.5% of patients were employed/admitted to educational programs (25). Improving treatment outcomes and quality of life of patients is a core focus of the program (24) and thus a systematic needs assessment complements the program’s aims. To the best of our knowledge, no studies have been conducted in an Asian early psychosis population to understand the different facets of patient needs. Such a study could be informative in improving patients’ care experience.

This study aimed to understand the (a) needs felt by patients and those perceived by CPs, (b) agreement between patients and CPs in the identified needs, and (c) factors associated with unmet needs.

## Materials and methods

### Participants and setting

The study recruited patients from the outpatient clinics of EPIP. Eligible patients were aged 18–40 years, had a diagnosis of schizophrenia spectrum and other psychotic disorder, had sufficient mental capacity to participate in the study as assessed by the clinician or trained study team member, and were able to read and converse in English. The language of the survey was restricted to English as the majority of EPIP patients are young adults or adolescents who can speak English. The patients were asked to nominate three CPs who according to them knew their care needs well. The CPs were approached in the order of preference, as indicated by the patient. Patients who were in the acute phase (within 3 months from the first visit to EPIP), with a diagnosis other than schizophrenia spectrum and other psychotic disorder, unable to provide consent [and parental consent for minors (below 21 years)] were excluded. Those in the acute phase (3 months of their first visit to EPIP) were excluded as they are clinically unstable which affects their capacity to provide informed consent and answer the questions in a thoughtful manner.

Patients were recruited through referrals by their attending care teams. A trained team member explained the study and took written informed consent from the patient (and parent, for minors), before administering the survey either face to face or through videoconferencing. All, except the need assessment questionnaire were self-administered. The study followed procedures approved by the Institutional Research Review Committee and Domain Specific Review Board (DSRB Ref: 2020/01112).

### Sample size

The sample size was estimated based on the results from Uygur and Danaci (12). To estimate the needs of patients with schizophrenia spectrum and other psychotic disorders using similar instruments with a desired width of the confidence interval or precision set at 1 and confidence level set at 95%, a sample size from 53 to 87 was required. After considering a withdrawal rate of approximately 20%, the study required a total sample size of 104 patients and their CPs. The patients and the CPs were recruited in 1:1 dyad and thus, the desirable total sample size of 208 was estimated.

### Measures

#### Sociodemographic information

Age, gender, ethnicity, marital status, income, and employment status were captured through a structured questionnaire.

#### Clinical variables

Number of hospital admissions (“In the past 12 months have you been admitted to the hospital for mental health issues”?) and psychiatric medications over the past 12 months (Yes/No) were captured through self-report while Positive and Negative Syndrome Scale (PANSS), treatment adherence [medication adherence: assessed by the CP as non-adherent (0–49%; partial adherence: 50–74%; adherent: 75–100%)], duration of untreated psychosis (onset of symptoms to first visit to EPIP, dichotomized as more than 1 year or less than 1 year), and treatment duration which was calculated based on the duration from first visit to the date of interview (dichotomized as less than 1 year or more than 1 year), were captured from the EPIP clinical database.

#### Needs assessments

Camberwell Assessment of Need Short Appraisal Version (CANSAS) assessed needs in 5 domains corresponding to 22 different areas/subdomains including (a) basic needs (accommodation, food, daytime activities) (b) health [physical health (needs help with physical health conditions), psychotic symptoms (needs care to alleviate psychotic symptoms), psychological distress (feeling sad, anxious or frightened), safety to self and others, alcohol, (abuse of) drugs), (c) functioning (self-care, looking after the home, child care, education, and money) (d) social (company (social life), intimate relationships (relationship with partner), and sexual expression (sexual practices and contraception)] (e) services [information on the conditions and its treatment, transportation, telephone, and benefits (other entitlements)]. The responses were captured on a 4-point scale (“no need,” “met need,” “unmet need” and “unknown need”). The ratings were captured by trained study team members as per the guidelines (26). The scale had been previously validated in mental health populations (16, 27).

#### Depression

Depressive symptoms were assessed using Patient Health Questionnaire-9 (PHQ-9), a validated 9-item scale scored on a 4-point scale [Not at all (0), several days (1), more than half the days (2), nearly every day (3)]. A score of 0–4 indicates no/minimal depression, 5–9 is mild, 10–14 is moderate, 15–19 is moderately severe and 20–27 indicates severe depression (28). Due to the small frequencies within the categories, regrouping was done as follows for the regression analysis: none to minimal, mild, moderate, and moderately severe to severe.

#### Anxiety

Generalized Anxiety Disorder (GAD-7) scale is a validated tool to assess Generalized anxiety disorder in clinical practice and research. The 7- items scale captures the responses on a 4-point scale (“not at all,” “several days,” “more than half the days,” and “nearly every day”). The total score ranges from 0 to 21. A score of 5 represents a mild level of anxiety and 10 and 15 represents moderate, and severe levels, respectively (29). The categories were regrouped as minimal, mild-moderate, and severe for the regression analysis due to the small sample within the categories.

#### Obsessive-compulsive disorder

Obsessive-Compulsive Inventory-Revised (OCI-R) was used to capture OCD. It is an 18 items tool where responses are captured on a 5-point scale (“not at all,” “a little,” “moderately,” “a lot,” “extremely”) to evaluate 6 groups of OCD symptoms (washing, checking, ordering, obsessing, hoarding, and neutralizing). The total score ranges from 0 to 72. A cut-off score of 21 identifies 66% of OCD cases while a cut-off score of 5 identifies 99.5% cases. The scale has been validated in several studies (30, 31).

### Analysis

A descriptive analysis was performed on categorical variables such as sociodemographic information which is expressed as frequency and percentages. The CANSAS scores between the groups (patient vs. CP) were compared using paired *t*-test. The agreement between CP and patients were assessed using Cohen’s Kappa coefficients. Kappa values above 0.80 were considered strong agreement while 0.61–0.80 indicates substantial agreement, 0.41–0.60 indicate moderate agreement, 0.21 to 0.40 indicates fair agreement and below 0.20 indicate slight agreement (32). Differences in the ratings of unmet needs between patients and CPs were calculated using paired sample *t*-test. Factors associated with unmet needs in patients were calculated using linear regression using unmet needs as outcome variable and sociodemographic and clinical variables as independent variables. A univariate analysis was initially performed and the variables that showed significant differences were included in the linear regression. All the analyses were done using IBM SPSS statistics version 25 (IBM SPSS statistics for Windows, Version 23.0, Armonk, NY: IBM Corp.).

## Results

### Sociodemographic characteristics of the participants

Table 1 shows the sociodemographic characteristics of the participants. A total of 109 patients and 106 CPs were recruited leading to 106 pairwise comparison of needs. The mean age of patients and CPs were 27.13 (±5.4) and 36.44 (±8.08) years, respectively. Around 65% of patients were Chinese, 62.39% had an education status of tertiary or above and 45.9% were employed, whilst 67.92% of CPs were females and had 9.5 years (median) of work experience in the current setting. Around 54% of patients were not admitted to the hospital in the past year and 93.58% were on medications (past 12 months). Around 72.48% were attending treatment for more than a year and 75.23% reported a duration of less than a year of untreated psychosis. Around 49.54% were adherent to treatment, 47.7% had depression (mild to severe), 33.9% had anxiety (mild to severe), and 12.8% met the clinical threshold of OCD.

**TABLE 1:** Sociodemographic characteristics of the participants.

	Patients (*n* = 109)		Care providers (*n* = 106)	
		
Sociodemographic variables	*n*	%	*n*	%
**Age**				
**Mean (SD)**	27.13 (± 5.40)	−	36.44 (± 8.08)	−
**Median**	27.00	−	36.5	−
18–21	18	16.51	−	−
22–30	61	55.96	−	−
31–40	30	27.52	−	−
**Gender**				
Male	59	54.13	34	32.07
Female	50	45.87	72	67.92
**Ethnicity**				
Chinese	71	65.14	60	56.6
Malay	23	21.10	13	12.26
Indian	8	7.34	12	11.32
Others	7	6.42	21	19.81
**Religion**				
Christianity	28	25.69	34	32.08
Buddhism	21	19.27	0	0
Hinduism	4	3.67	11	10.38
Islam	27	24.77	27	25.47
Free thinkers	23	21.11	25	23.58
Others	6	5.50	9	8.49
**Education**				
Secondary or below (primary, secondary, pre-u/Junior College, Vocational Institute/ITE)	40	36.7%	0	−
Tertiary or above (diploma, degree, professional certification, and above)	68	62.39%	103	97.17
Missing	1	0.92		
**Marital status**				
Married/cohabiting	6	5.50	74	69.81
Others*	103	94.50	32	30.19
**Employment**				
Employed	50	45.90	−	−
Others (economically inactive, unemployed)	59	54.10	−	−
**Years of experience**				
**Institutional**				
Mean (SD)	−	−	8.58 (± 6.59)	
Median	−	−	9.50	
Overall	−	−		
Mean (SD)	−	−	10.23 (± 7.34)	
Median	−	−	10.00	
**Monthly income**				
Below 2,000	63	57.80	−	−
2,000 and above	32	29.36	−	−
Do not know or I don’t wish to answer	14	12.84	−	−
**Hospital admission (12 months)**				
Yes	49	44.95	−	−
No	59	54.13	−	−
Missing	1	0.92	−	−
**Psychiatric medication (12 months)**				
Yes	102	93.58	−	−
No	7	6.42	−	−
**Treatment duration**				
Less than 1 year	29	26.61	−	−
More than 1 year	79	72.48	−	−
Missing	1	0.92	−	−
**Duration of untreated psychosis**				
Less than 1 year	82	75.23	−	−
More than 1 year	23	21.10	−	−
Missing	5	3.67	−	−
**Treatment adherence**				
Adherent	54	9.17	−	−
Non-adherent	10	9.17	−	−
Partial adherent	8	7.34	−	−
Not on medication	2	1.83	−	−
Unknown	35	32.11	−	−
**Health Status**				
**PANSS score**				
PANSS positive (median)	7.00	−	−	−
PANSS negative (median)	7.00	−	−	−
PANSS total (median)	36.00	−	−	−
**Depression**				
None or minimal	57	52.29	−	−
Mild	26	23.85	−	−
Moderate	13	11.93	−	−
Moderately severe	10	9.17	−	−
Severe	3	2.75	−	−
**Anxiety**				
Minimal	72	66.06	−	−
Mild	18	16.51	−	−
Moderate	11	10.09	−	−
Severe	8	7.34	−	−
**OCD**				
Below clinical threshold	95	87.16	−	−
Above clinical threshold	14	12.84	−	−

*Unmarried, separated, widowed or divorced.

### Needs identified by patients and care providers

The average number of total needs identified were 4.06 and 3.84 for patients and CPs, respectively. Overall patients rated 0.3 more needs (out of 22) than CPs. The average unmet needs identified were 1.26 and 1.02 by patients and CPs, respectively (*p* ≥ 0.05).

The majority of patients reported absence of needs on all items, except the needs regarding psychotic symptoms, under the “health” domain (Table 2, 6.6%; *n* = 7 for patients compared to 14.15%; *n* = 15 for CPs), and on information about the disease and treatment under the “services” domain (50.94%; *n* = 54 of patient vs. 68.87%; *n* = 73 of CPs). For “psychotic symptoms,” the majority of patients who reported a need indicated the need as “met” (83.02%; *n* = 88 of patients compared to 77.36%; *n* = 82 of CPs). For “information on disease and treatment,” 41.51% (*n* = 44) and 7.55% (*n* = 8) of the patients had met and unmet needs, whilst 30.19% (*n* = 32) and 0% of CPs identified a met and unmet need, respectively. The highest number of unmet needs were identified for the social (50% of patients and CP) and health domains (31.13% of patients vs. 28.30% by CP).

**TABLE 2:** Needs endorsed by the patients and CPs and their concordance.

	Patient, *n* (%)				Care provider, *n* (%)				
			
	No need	Met	Unmet	Not known	No need	Met	Unmet	Not known	Cohen’s kappa*
**Basic**									
Accommodation	102 (96.23)	3 (2.83)	1 (0.94)	0 (0)	95 (89.62)	9 (8.49)	2 (1.89)	0 (0)	0.23
Food	99 (93.40)	7 (6.60)	0 (0)	0 (0)	94 (88.68)	11 (10.38)	0 (0)	1 (0.94)	0.03
Day time activities	89 (83.96)	8 (7.55)	9 (8.49)	0 (0)	83 (78.30)	10 (9.43)	13 (12.26)	0 (0)	0.27
**Health**									
Physical health	84 (79.25)	16 (15.09)	6 (5.66)	0 (0)	66 (62.26)	23 (21.70)	14 (13.21)	3 (2.83)	0.19
Psychotic symptoms	7 (6.60)	88 (83.02)	11 (10.38)	0 (0)	15 (14.15)	82 (77.36)	8 (7.55)	1 (0.94)	0.20
Psychological distress	65 (61.32)	32 (30.19)	9 (8.49)	0 (0)	67 (63.21)	32 (30.19)	6 (5.66)	1 (0.94)	0.31
Safety to self	91 (85.85)	11 (10.38)	4 (3.77)	0 (0)	96 (90.57)	9 (8.49)	1 (0.94)	0 (0)	0.25
Safety to others	98 (92.45)	5 (4.72)	3 (2.83)	0 (0)	101 (95.28)	4 (3.77)	1 (0.94)	0 (0)	0.03
Alcohol	106 (100.00)	0 (0)	0 (0)	0 (0)	98 (92.45)	1 (0.94)	1 (0.94)	6 (5.66)	•
Drugs	106 (100.00)	0 (0)	0 (0)	0 (0)	102 (96.23)	1 (0.94)	0 (0)	3 (2.83)	•
**Social**									
Company	63 (59.43)	18 (16.98)	25 (23.58)	0 (0)	61 (57.55)	11 (10.38)	32 (30.19)	2 (1.89)	0.40
Intimate relationships	81 (76.42)	5 (4.72)	18 (16.98)	2 (1.89)	59 (55.66)	6 (5.66)	19 (17.92)	22 (20.75)	0.18
Sexual expression	92 (86.79)	0 (0)	10 (9.43)	4 (3.77)	39 (36.79)	1 (0.94)	2 (1.89)	64 (60.38)	0.04
**Functioning**									
Looking after the home	90 (84.91)	13 (12.26)	3 (2.83)	0 (0)	97 (91.51)	6 (5.66)	2 (1.89)	1 (0.94)	0.13
Self-care	95 (89.62)	7 (6.60)	4 (3.77)	0 (0)	91 (85.85)	13 (12.26)	1 (0.94)	1 (0.94)	0.19
Child care (dependents)	103 (97.17)	3 (2.83)	0 (0)	0 (0)	104 (98.11)	1 (0.94)	1 (0.94)	0 (0)	0.59
Education	100 (94.33)	3 (2.83)	3 (2.83)	0 (0)	102 (96.23)	4 (3.77)	0 (0)	0 (0)	0.07
Money	72 (67.92)	23 (21.70)	10 (9.43)	1 (0.94)	76 (71.70)	20 (18.87)	5 (4.72)	5 (4.72)	0.21
**Services**									
Information	54 (50.94)	44 (41.51)	8 (7.55)	0 (0)	73 (68.87)	32 (30.19)	0 (0)	1 (0.94)	0.35
Transport	101 (95.28)	4 (3.77)	1 (0.94)	0 (0)	99 (93.40)	6 (5.66)	1 (0.94)	0 (0)	0.13
Telephone	103 (97.17)	2 (1.89)	1 (0.94)	0 (0)	100 (94.34)	6 (5.66)	0 (0)	0 (0)	0.08
Benefits	84 (79.25)	8 (7.55)	8 (7.55)	6 (5.66)	89 (83.96)	11 (10.38)	2 (1.89)	4 (3.77)	0.24

*Kappa values above 0.80 were considered strong agreement while 0.61–0.80 indicates substantial agreement, 0.41 to 0.60 indicate moderate agreement, 0.21–0.40 indicates fair agreement and below 0.20 indicate slight agreement. • Kappa could not be computed due to insufficiently spread data.

While the number of participants (patients and CPs) who identified unmet needs were similar for “social” and “health” domains, a higher number of CPs (14.15%; *n* = 15) when compared to the patients (9.43%; *n* = 10) identified unmet needs in “basic” needs domain. On the contrary, a higher number of patients identified unmet needs for “functioning” (18.87%; *n* = 20 vs. 8.49%; *n* = 9) and “services” (16.98%; *n* = 18 vs. 2.83%; *n* = 3) when compared to the CPs. CPs perceived higher number of unmet needs on 4 specific items under various domains when compared to the patients: accommodation (1.89% vs. 0.94%), company (30.19% vs. 23.58%), alcohol (0.94% vs. 0%) and intimate relationships (17.92% vs. 16.98%). Company, intimate relationships, psychotic symptoms, money, sexual expression, psychological distress, information, and benefits were the most frequent unmet needs identified by the patients. On the other hand, company, intimate relationships, physical health, and daytime activities were the unmet needs identified by CPs.

Highest number of needs were identified (met or unmet needs) for health, social and services domain (Supplementary Table 1).

### Concordance between patients and care providers

Moderate agreement (*k* = 0.41–0.6, Table 2) was noted only for the childcare/dependents needs (*k* = 0.59). Fair agreement (*k* = 0.21–0.4) was noted for basic needs (accommodation, daytime activities), health (psychological distress, safety to self), social (company), functioning (money) and for services (information, benefits). Slight agreement (*k* = 0–0.2) was noted for services (transport, telephone) functioning (looking after home, self-care and education), health (physical health, psychotic symptoms, safety to others) and basic needs (food). The lowest level of agreement was noted for needs for food (*k* = 0.03), safety to others (*k* = 0.03), sexual expression (*k* = 0.04), education (*k* = 0.07) and telephone (*k* = 0.08). Among the items identified as unmet needs, company and intimate relationship were the top 2 items endorsed by the patients and CPs in agreement which each other.

### Factors influencing unmet needs felt by the patients

Duration of untreated psychosis, PANSS score, treatment adherence, hospital admissions, psychiatric medication and treatment duration showed no significant relationship with total unmet needs. Age, depression, anxiety, and OCD showed a positive association with unmet needs. Compared to minors (18–21 years old), adults (aged 31–40 years) tend to endorse significantly more unmet needs (B: 1.10; 95% CI: 0.15–2.05; *p* = 0.03; Table 3). Those with moderate severity of depression tend to report higher number of unmet needs (B: 1.35; 95% CI: 0.35–2.35; *p* = 0.01) compared to those with no or minimal depressive symptoms. Similarly, those with severe anxiety (B: 2.18; 95% CI: 0.52–43.84; *p* = 0.01), and OCD (B: 1.69; 95% CI: 0.61–2.76; < *p* = 0.01) reported more unmet needs than those with minimal anxiety and below clinical threshold OCD, respectively.

**TABLE 3:** Results of linear regression examining the sociodemographic and clinical correlates of needs felt by patients.

	Total unmet needs			
	
	B	95% CI		*p*-value
**Independent variables**				
**Age**				
18–21	Ref			
22–30	0.35	–0.47	1.17	0.40
31–40	1.10	0.15	2.05	**0.03**
**Gender**				
Male	Ref			
Female	–0.40	–0.96	0.17	0.16
**Ethnicity**				
Chinese	Ref			
Malay	–1.13	–3.18	0.92	0.28
Indian	–1.10	–2.63	0.44	0.16
Others	–0.11	–1.68	1.47	0.89
**Religion**				
Free thinkers	Ref			
Christianity	0.43	–0.41	1.26	0.31
Buddhism	–0.03	–0.88	0.83	0.95
Hinduism	1.40	–0.73	3.53	0.19
Islam	1.35	–0.66	3.36	0.18
Others	0.64	–0.93	2.20	0.42
**Employment**				
Employed	Ref			
Others (Economically inactive, unemployed	0.54	–0.06	1.13	0.08
**Depression**				
None or minimal	Ref			
Mild	0.07	–0.62	0.75	0.84
Moderate	1.35	0.35	2.35	**0.01**
Moderately severe to severe	–1.23	–2.76	0.29	0.11
**Anxiety**				
Minimal	Ref			
Mild to moderate	0.10	–0.66	0.87	0.80
Severe	2.18	0.52	3.84	**0.01**
**OCD**				
Below clinical threshold	Ref			
Above clinical threshold	1.69	0.61	2.76	**< 0.01**

Bold values denote statistically significant B value.

## Discussion

In the current study, patients and CPs identified similar need domains with an average of 4.06 and 3.84, needs, respectively. Both the groups identified health and social domains to have a higher number of unmet needs. The agreement between patients and CPs were, however, low. Age, depression, anxiety, and OCD were positively associated with unmet needs felt by patients.

In the current study, patients and CPs identified an average of 4.06 and 3.84 needs, respectively. Patients endorsed 1.26 unmet needs compared to 1.02 by CPs. This is lower than what was reported by Slade et al. (27) who reported 6.7 and 6.1 needs for patients and CPs, respectively. However, it is similar to Lasalvia et al. (33) who observed an average need endorsement of 3.64 in a sample of patients with psychosis in Italy. However, the authors did not analyze the average unmet needs in their study. Jorquera et al. (34) identified an average of 1.0 unmet needs per patients which is in agreement with our results. Fleury et al. (35) compared the needs endorsed by participants from different areas in Quebec and showed that metropolitan areas were vulnerable to unmet needs. The authors also compared the mental health needs endorsed by various studies and showed a varying level of need endorsements ranging from 3.07 to 9.5 by patients and 2.4–10.9 by CPs. McCrone et al. (20) compared the needs of schizophrenia patients across the world and concluded that the needs are different across countries. These observed differences were proposed to be due to area specific factors, interviewer characteristics (interviewers who are clinicians are well acquainted to the needs and thus will identify more problems) or cultural differences (20, 35). Contrary to that reported in developed nations, studies conducted in developing nations showed different domains of unmet needs (26). A study conducted in Brazil among patients with early psychosis identified transportation and benefits as domains of unmet needs (36). This was attributed to the lack of awareness of the participants regarding their benefit entitlements. A similar study in Ethiopia among individuals with psychosis identified higher unmet needs on basic (accommodation, food, daytime activities) and social life domains (37). Thus, the needs of the patients could be different based on the economic status, infrastructure, and healthcare policies. The lower endorsement of needs in the current study could be attributed to the local services. Singapore being a developed country that has an efficient healthcare system and community level outreach could have addressed the basic growth and deficiency needs through strong policies that support the needs of the population.

The majority of participants endorsed having no need for most of the items on the scale except for specific items (psychotic symptoms and information on condition and treatment), for which a substantial proportion from both the groups endorsed that the needs are met (psychotic symptoms: 83.02%; information: 41.51%). This is probably because the treatment is delivered specifically for the psychotic symptoms and thus the treatment needs for psychotic symptoms and need for information is fulfilled for the majority of patients who are seeking treatment for first episode psychosis 39). This agrees with previous reports (12, 34, 38). Endorsement of a fulfilled need (met need) is captured as the presence of a need for which the patient has received help from the health care system, family/friends, or community partners to fulfill the need. Thus, it can be considered as a positive outcome of treatment. EPIP Singapore has an efficient patient management system that involves a multidisciplinary team assessing and taking care of the needs of the patients. Thus, the low endorsement of unmet needs could be a reflection of care provided by the program. Nonetheless, Jorquera et al. (34) have warned that emphasis on clinical elements alone often leads to the unintentional neglect of other domains that are of paramount importance in achieving the desired treatment outcomes.

Needs (met or unmet) were reported for “social,” “health” and “services” domains by both patients and CPs with the former two being the domains of most of the unmet needs. In particular, company (social interaction), intimate relationships, psychotic symptoms, money, sexual expression and psychological distress, information and benefits were identified to have an unmet need in descending order with the former three being the most prominent unmet needs felt by the patients. EPIP offers a system which addresses the basic, social, services, functioning and healthcare needs of the patients. Nonetheless, the pandemic had a tremendous impact on the care delivery during the past 2 years where social distancing measures and healthcare burden of the pandemic had affected group therapy sessions and face to face consultations that were meant to address these aspects. The data was captured during this period where there were ongoing restrictions that prevented social interactions between CPs and patients or among patients. Thus, the unmet needs reflected in the social and services domains could reflect the pandemic induced barriers to care that included inability to hold face to face sessions such as group activities or group therapy, psychoeducation and other social activities which are a part of the treatment. It could also be attributed to illness specific factors such as negative symptoms e.g., avolition and social withdrawal which are commonly observed in schizophrenia. Furthermore, as the needs of the patients change at different stages of their life/disease (39, 40) it is possible that the needs emerged during the course of their treatment journey. Therefore, an ongoing evaluation of the needs must be integrated to the psychoeducation programs to address emerging needs. It is also possible that the patients often forget the information on their treatment and disease given by their care team and thus continued education/communication as a part of the existing program would be a useful strategy to mitigate the unmet needs. We also noted unmet needs in sexual expression (sexual life and contraception) and intimate relationships. In Asian culture, sexual expression and relationships are considered as extremely private topics which are not often discussed between patients and CPs due to embarrassment, cultural influences, or lack of training of the CPs to handle these sensitive topics. Nonetheless, unmet needs in these domains requires attention and the care team should work toward identifying the causes of the unmet needs to take effective steps to resolve them.

A few of the studies have reported similar domains of unmet needs (41), however, the domains and items identified in various studies tend to differ. Psychological distress was a common unmet need identified in most of the studies (12, 27, 34, 35), as it is a common occurrence in recovering patients who understand the reality of their condition once their symptoms stabilize. They feel the stress, worry, grief, or anxiety with their social situation and reintegration which stems from their social exclusion due to the mental illness or lack of social skills. Other studies conducted in schizophrenia patients identified mostly different unmet needs (12, 27, 34, 35). A systematic review of 20 studies showed that social relationships, information about disease and psychological distress were the main sources of unmet needs in people with mental illness (5). The source of these discrepancies is multifactorial and could be attributed to the sociocultural context/characteristics of the sample and the differences in the care systems (8). Socioeconomic status, social contact, family relationship and stigmatization in the community influences the type of needs felt by the patients (8). Another factor that could play a key role in the observed discrepancy among studies is the severity and stage of the disease. A study conducted in Australia among people with mental illness showed higher unmet needs for company, daytime activity and intimate relationship in community and inpatient samples (42). The authors noted that the need was higher for inpatient samples. In contrast, long stay patients with a psychotic disorder reported unmet needs on domains for accommodation, social and care (43). Also, the needs of the patients evolve with time and are different at different time points (39, 40). Unmet needs are also dependent on the stage of the disease. Although the available evidence in early psychosis patients is scarce, it was noted that psychotic symptoms, psychological distress, daily activities and social relationships were the needs endorsed by the patients with early psychosis, which is similar to our observation (34, 40). These reports thus emphasize the importance of needs assessments at regular intervals in specific subpopulations of diagnoses to capture the evolving needs of the patients in the context of the limitations in applying the results across populations.

We noted low levels of agreement ranging from slight to fair between the CP and patients in the majority of domains (19 out of 22). This could be due to various reasons which include staff characteristics where CPs with different professional expertise focus on specific needs thus some needs are inadvertently ignored. The underpinnings of this omission could be the differences in the perception of necessity, convenience, lack of knowledge or inability to reach a consensus even after the discussion (32). Our CPs identified by the patients included psychiatrists (*n* = 24), case managers (*n* = 77), psychologists (*n* = 3) and peer support specialist (*n* = 2) who have different level of expertise and interactions with the patients. Similar results were recorded in previous studies (32). While the staff might be aware of the clinical care needs, they might not have sufficient knowledge on other domains, leading to a low concordance (27, 32, 35, 44). Fleury et al. (35) looked at CP specific characteristics and showed its influence on the needs identified. Psychiatrists tend to identify only clinical issues while social workers and other professionals endorse problems in other social, functioning and service-related domains Nonetheless, the lack of a substantial concordance between the patients and CPs has clinical implications as it reflects a lack of understanding of actual unmet needs which would have an impact on the treatment process and its outcome. Thus, an approach that involves training of the CPs to identify various areas of needs of their patients beyond the clinical elements and effective planning to monitor the unmet needs observed in areas with slight agreement (services, functioning and health domains) along with strategies to fulfill the needs could achieve favorable outcomes.

We have also noted that sociodemographic and clinical characteristics of the patients were associated with the unmet needs. Older adults (31–40 years) when compared to young adults (18–21 years) reported significantly higher number of unmet needs. This could be attributed do their family environment, socioeconomic status, and comorbidities. It is possible that the older age group had higher commitments than the younger age group and thus report higher unmet needs. Also, it is well known that older patients are more prone to chronic conditions that could add on to their unmet needs. Similar results were reported previously (45, 46). We have noted that those with moderate depressive symptoms, OCD and severe anxiety tend to report significantly higher number of unmet needs. Other studies have also noted similar findings (12, 47). Depression, anxiety, and OCD are highly prevalent in those with schizophrenia spectrum and other psychotic disorders. Our previous studies have noted a prevalence of 16.5% for depression and 5.1% for OCD in the EPIP population (48) which could lead to unmet needs, especially in psychiatric and psychosocial domains in patients with early psychosis (20, 49, 50). We did not observe a relationship between the treatment duration, adherence to medication and duration of untreated psychosis with unmet needs. While our results corroborate the findings by Landolt et al. (40) who studied the correlates of unmet needs in early psychosis and reported no relationship between these variables, it contradicts the findings from Jorquera et al. (34). This could be attributed to the same characteristics that were discussed earlier.

This study was conducted in a sample of patients receiving care for first episode psychosis and thus adds value to the knowledge gaps on the needs of this population. We have also considered the treatment duration, adherence, duration of untreated psychosis, use of medication and hospitalization to account for the factors that could affect the unmet needs. The sample was drawn from the outpatient clinic of the EPIP and thus the results cannot be generalized without reservation. Also, we have employed the kappa coefficients to deduce the concordance between patients and CPs. While kappa coefficients were calculated due to its advantage of controlling for expected agreement levels occurring by chance, it should still be interpreted with caution, especially when obvious disparity in needs are observed between parties of interest. Finally, the low coefficient is not exclusively an indication of the low agreement but could also be due to the skewed distribution of the values across the categories. Future research should focus on gaining an in depth understanding of the reasons for unmet needs reported in specific domains and patients’ perspective/suggestions to overcome these unmet needs. A qualitative study would shed light on this unknown aspect and give directions for improving the consensus of unmet needs between patients and CPs. Furthermore, additional domains of needs that are relevant in early psychosis patients such as, cognitive functions (memory), and needs for occupational/role functioning (attaining goals/aspiration, independent work/studies) should be explored to ensure holistic care.

## Conclusion

Highest number of unmet needs were identified by patients and CPs in social, health and functioning domain with patients identifying highest unmet needs in functioning and services domain. The items that scored the highest proportion of unmet needs were company, intimate relationships (social domain) followed by psychotic symptoms (health domain). The agreement between patients and CPs were slight to-fair. Low concordance could reflect the lack of knowledge of the CPs in that particular domain stemming from lack of expertise, or disagreements between patients and CPs and should be improved through effective discussion and resolution of the problems. Older age, having depressive symptoms, OCD and severe anxiety were associated with unmet needs felt by the patients. Treatment of psychiatric comorbidities could alleviate some of the unmet needs. Routine monitoring of the needs of patients could reveal changing perspectives and dynamics that will enable CPs to work toward fulfilling the unmet needs through effective communication and goal setting. Such arrangements could improve treatment outcomes and quality of life of patients.

## Data availability statement

The original contributions presented in this study are included in the article/Supplementary material, further inquiries can be directed to the corresponding author.

## Ethics statement

The studies involving human participants were reviewed and approved by the National Healthcare Group’s Domain Specific Review Board (DSRB Ref:2020/01112). Written informed consent to participate in this study was provided by the participant or his/her legal guardian (if minor) legal guardian/next of kin.

## Author contributions

PA and MS conceptualized the study. PA, MS, ES, SV, CT, SK, and LZ contributed to study methodology. PA, PS, PW, YT, SV, CT, JH, LC, and ES were involved in the data collection. ES and PA performed data validation and quality checks. PA, MS, and YT performed data analysis and interpretation. PA wrote the first draft of the manuscript. All authors revised the manuscript and approved the final manuscript.
