# Supplementary material for: Needs of patients with early psychosis: A comparison of patient’s and mental health care provider’s perception
Source: Front Psychiatry. 2022 Sep 20;13:952666. doi: 10.3389/fpsyt.2022.952666 (PMC9531114; doi:10.3389/fpsyt.2022.952666)
Supplement: Supplementary file 1 [file Table_1.DOCX]

Supplementary Material

# Supplementary Data

**Supplementary table 1:** Total needs identified by the participants

|  | **Patient identifying a need n (%)** | **Care provider identifying a need n (%)** | **Patient/Staff pairs identifying a need n (%)** |
| --- | --- | --- | --- |
| **Basic** |  |  |  |
| Accommodation | 4 (3.77) | 11 (10.38) | 2 (1.89) |
| Food | 7 (6.60) | 11 (10.38) | 1 (0.94) |
| Day time activities | 17 (16.04) | 23 (21.69) | 6 (5.66) |
| **Health** |  |  |  |
| Physical health | 22 (20.75) | 37 (34.91) | 9 (8.49) |
| Psychotic symptoms | 99 (93.40) | 90 (84.91) | 73 (68.87) |
| Psychological distress | 41 (38.68) | 38 (35.85) | 18 (16.98) |
| Safety to self | 15 (14.15) | 10 (9.43) | 3 (2.83) |
| Safety to others | 8 (7.55) | 5 (4.71) | 0 (0) |
| Alcohol | 0 | 2 (1.88) | 0 (0) |
| Drugs | 0 | 1 (0.94) | 0 (0) |
| **Social** |  |  |  |
| Company | 43 (40.57) | 43 (40.57) | 21 (19.81) |
| Intimate relationships | 23 (21.70) | 25 (23.58) | 8 (7.55) |
| Sexual expression | 10 (9.43) | 3 (2.83) | 1 (0.94) |
| **Functioning** |  |  |  |
| Looking after the home | 16 (15.09) | 8 (7.55) | 2 (1.89) |
| Self-care | 11 (10.37) | 14 (13.20) | 2 (1.89) |
| Child care (Dependents) | 3 (2.83) | 2 (1.88) | 1 (0.94) |
| Education | 6 (5.66) | 4 (3.77) | 0 (0) |
| Money | 33 (31.13) | 25 (23.59) | 10 (9.43) |
| **Services** |  |  |  |
| Information | 52 (49.06) | 32 (30.19) | 23 (21.70) |
| Transport | 5 (4.17) | 7 (6.60) | 1 (0.94) |
| Telephone | 3 (2.83) | 6 (5.66) | 0 (0) |
| Benefits | 16 (15.10) | 13 (12.27) | 4 (3.77) |
